# Editorial Notes

**Published:** 1893-09

**Authors:** 


### Editorial Notes.

Dr. L. M. Crow has moved to Athens, Ga.

Dr. William H. Cumming died in Marietta, Ga., on 21st.

Dr. K. P. Moore, of Macon, Ga., is doing the World's Fair.

Dr. Frank M. Ridley, LaGrange, Ga., called on us a few days ago.

Dr. James W. Camak, of Athens, Ga., died last week, in the above city.

Dr. W. N. Judson, of this city, died at Indian Springs, August 18th.

Dr. J. L. Stockdell has moved to this city. Office in Equitable Building.

Dr. R. D. McLeod, of Lyons, Ga., is spending a few weeks in the city visiting friends.

MEDICAL SOCIETY OF VIRGINIA.—The 24th annual session will convene in Charlottesville, Va., October 3d.

American Electro-Therapeutic Association will hold its 31st annual meeting in Chicago, Ill., September 12th, 13th and 14th.

Dr. J. Allison Hodges, of Wilmington, N. C., has been elected Professor of Anatomy in the College of Physicians and Surgeons, Richmond, Va.

Dr. R. S. Woodson, of the United States Army, came up from Florida, where he has been stationed with the United States troops for some time past. The army has no brighter or better surgeon than Dr. Woodson, who is well known in Atlanta, where he was once stationed.

Drs. A. J. Calloway, of Milledgeville, A. F. Harrington, of West Point, and Robert B. Cloud, of Gainesville, paid the RECORD pleasant calls last week.

---

Dr. T. L. Lallersted, of Panola, Ga., died last week. He was the leading physician of his county. The Medical Association looses one of its best members.

---

"THE 5th Annual Meeting of the Tri-State Medical Society of Alabama, Georgia and Tennessee, will be held in Chattanooga, Tuesday, Wednesday and Thursday, October 17th, 18th and 19th.

---

Dr. W. J. Blalock, who, having recently completed his medical education at the College of Physicians and Surgeons,in New York has settled in Atlanta and secured an office in the new Inman building on Broad street.

---

The Transactions of the Medical Society of the State of California, of the Session of 1893 is at hand. The book is neatly gotten up, and reflects credit on the Association and its Secretary, Dr. Wm. Watt Kerr.

---

We have just received the Transactions of the Medical Society of the State of New York for 1893. The volume is replete with papers of high merit and the binding and typographical work is excellent. Dr. Frederick C. Curtis, of Albany, is the efficient Secretary.

---

We are in receipt of the first issue of the *Journal of Surgery, Gynecology and Obstetrics*, published in this city and edited by Dr. C. Evans Johnson. The *Journal* has a host of collaborators who no doubt will make its pages gleam with articles of great interest. Dr. Johnson is a young gentleman of boundless energy and pluck and the RECORD desires to congratulate him upon the neat appearance of his journal. We wish it unbounded success.

Dr. A. G. Hobbs will attend the Pan-American Medical Congress at Washington, on September 6th. He will read a paper before the Congress entitled, "The Simplest and Most Practical Electric Apparatus, Especially for Galvano-cautery Operations." He has been appointed to discuss the following papers: "Injudicious Nasal Operations." "Sextons Operation for Excision of the Ossicles." "The Want of Co-ordination of the Ocular Muscles," and other subjects.

---

The American Medical Editors will have a meeting and banquet in Washington on the evening of Monday, September 4th, the day preceding the assembling of the Pan-American Medical Congress.

Dr. I. N. Love, of the *Medical Mirror*, 3642 Lindell Avenue, St. Louis, has been appointed Chairman of the Committee of Arrangements for Banquet, which fact gives ample assurance of the success of the latter.

It is earnestly hoped that every medical editor of all of the Americas will endeavor to be present on the interesting occasion. Please address the Chairman of Committee of Arrangements promptly.

---

AMERICAN SURGICAL ASSOCIATION.—At the recent meeting of the American Surgical Association the following were elected as the officers for the ensuing year: President, Dr. J. Ewing Mears, of Philadelphia; first Vice-President, Dr. Roswell Park, of Buffalo; second Vice-President, Dr. Lewis S. Pilcher, of Brooklyn; Secretary, Dr. J. R. Weist, of Richmond, Ind.; Treasurer, Dr. John B. Roberts, of Philadelphia; Recorder, Dr. DeForest Willard, of Philadelphia; Member of Council, Dr. J. Collins Warren, of Boston; Chairman of Committee of Arrangements, Dr. L. McLane Tiffany, Baltimore. The following were elected to membership: Drs. H. L. Burrell, of Boston; Perry H. Millard, of St. Paul; Albert B. Miles, of New Orleans; Samuel J. Mixter, of Boston; John W. Elliott, of Boston; John Parmenter, of Buffalo; J. McF. Gaston, of Atlanta. To honorary membership, Prof. Carl Gussenbauer, of Prague.

